# Parenteral Vaccine-Induced Antibody Levels in Children with Heavy Antibiotic Exposure in Dhaka, Bangladesh

**DOI:** 10.4269/ajtmh.25-0511

**Published:** 2026-06-04

**Authors:** Sarah B. Kohl, Dorothy M. Dickson, Masud Alam, E. Ross Colgate, William A. Petri, Rashidul Haque, Fiona van der Klis, Beth D. Kirkpatrick, Benjamin Lee

**Affiliations:** ^1^ Larner College of Medicine, University of Vermont, Burlington, Vermont, USA;; ^2^ Translational Global Infectious Disease Research Center, Larner College of Medicine, University of Vermont, Burlington, Vermont, USA;; ^3^ Division of Infectious Disease, Parasitology and Emerging Infections Laboratory, International Centre for Diarrhoeal Disease Research, Dhaka, Bangladesh;; ^4^ Division of Infectious Diseases & International Health, School of Medicine, University of Virginia, Charlottesville, Virginia, USA;; ^5^ Netherlands National Institute for Public Health and the Environment, Bilthoven, The Netherlands

## Abstract

Infants in resource-limited areas frequently receive early-life antibiotic treatment for respiratory and diarrheal illness, but the influence of antibiotics on parenteral vaccine immunogenicity in these settings remains underexplored. Therefore, we conducted a retrospective cohort study within a randomized, controlled, oral rotavirus and poliovirus vaccine trial performed in Dhaka, Bangladesh. Among 582 evaluable infants at 53 weeks of age, plasma antibody concentrations against measles, pertussis, diphtheria, tetanus, and *Haemophilus influenzae* type b (Hib) were evaluated for associations with prior antibiotic exposure. Most antibiotic prescriptions were broad-spectrum, with a mean of 85.5 days of antibiotics in the first year. Most children demonstrated protective antibody levels against measles (96%), tetanus (88%), and Hib (91%) at 53 weeks of age; fewer maintained protective levels against pertussis antigens (25–42%) and diphtheria (28%). Antibiotic exposure through 14 weeks of age, before completion of the primary vaccination series, did not significantly affect antibody concentrations at 53 weeks of age against diphtheria, tetanus, pertussis, and Hib antigens. Antibiotic exposure through 14 weeks of age was positively associated with measles antibodies at 53 weeks of age (change in concentration per antibiotic course was 9.09%; 95% CI: 5.13 to 12.75; *q*-value = 0.0003), but despite this association, nearly all children had protective antibody levels, limiting clinical significance. Although antibody levels varied by antigen, infant antibiotic exposure did not appear to clinically impact parenteral vaccine-induced antibody concentrations in this population. These findings support the robustness of parenteral vaccine responses in resource-limited settings despite high antibiotic use.

## INTRODUCTION

In resource-limited settings suffering from suboptimal community sanitation and hygiene, researchers often observe frequent antibiotic use in children in addressing higher incidences of respiratory and diarrheal disease.^1^ The main concern surrounding potential antibiotic overuse in this context has primarily centered on the emergence of antibiotic-resistant pathogens. However, the repercussions of excessive antibiotic administration may extend beyond the realm of antimicrobial resistance. Another major concern pertains to whether high rates of antibiotic therapy among the pediatric population could compromise parenteral vaccine immunogenicity.

Others have previously reported potential diminution of protective antibody levels in animal models and in children from the United States in association with increased antibiotic prescription and usage during the first 3 years of life.^2^^,^^3^ This effect was observed across various parenteral vaccines, including those targeting measles, pertussis, *Haemophilus influenzae* type b (Hib), tetanus, and diphtheria. However, there is a dearth of information investigating this phenomenon in populations from low- and middle-income countries (LMICs), where rates of antibiotic use during the first year of life are typically much higher. We sought to address this knowledge gap by investigating a pediatric population with an extremely high rate of antibiotic prescription in the first year of life.

We hypothesized that, as happens with children in the United States, the degree of antibiotic exposure during the first year of life would be associated with reductions in antibody levels following parenteral vaccination, which in turn might affect immune responses. In the Performance of Rotavirus and Oral Polio Vaccines in Developing Countries (PROVIDE) study conducted in Dhaka, Bangladesh, multiple factors were negatively associated with oral vaccine responses, as is typical for LMICs, including the likely co-occurrence of environmental enteric dysfunction (EED), a syndrome of subclinical intestinal inflammation; reduced absorptive capacity; and reduced barrier function believed to be common in LMICs due to unrelenting enteric pathogen exposure from fecal–oral contamination.^4^ Although oral polio vaccine responses were shown to be indirectly associated with antibiotic exposure via potential effects on T follicular helper cell function at age 2 years, significant differences in parenteral vaccine responses in the PROVIDE study were not detected, and a detailed exploration of antibiotic exposure across the spectrum of parenteral vaccine responses has not yet been performed.^5^

Therefore, we conducted a secondary analysis to evaluate associations between antibiotic use and parenteral vaccine-induced antibody levels in a cohort of infants from Dhaka, Bangladesh, where numerous antibiotic courses are common in the first year of life.

## MATERIALS AND METHODS

### Design.

The research was structured as a retrospective cohort study using data from the PROVIDE study, a birth cohort study conducted in Dhaka, Bangladesh, between 2011 and 2014. The PROVIDE study used a 2 × 2 factorial design to evaluate the clinical efficacy of the oral rotavirus vaccine Rotarix (GlaxoSmithKline; Philadelphia, PA) versus no vaccine at 10 and 17 weeks and the efficacy of a dose of inactivated polio vaccine (IPV) versus oral polio vaccine (OPV) at 39 weeks in inhibiting shedding of OPV at subsequent challenge at week 52. The study was approved by the ethical review boards of the International Centre for Diarrheal Disease Research, Bangladesh (icddr,b), the University of Vermont, and the University of Virginia. The study was registered at Clinicaltrials.gov (NCT01375647). Informed consent was obtained for all participating mothers and infants within 7 days after giving birth. Consent was also given for de-identified specimen retention and future research beyond the aims of the parent study. Seven hundred infants were enrolled and randomized in the first week of life and followed through 2 years of age; full study results from the parent study have been published.^6^^–^^8^

The study clinic served as the primary care office for most study participants, providing both primary and acute care services. Any concomitant medications, including new antibiotic treatments, prescribed by the study team were entered into the participant’s chart at the time of prescription. All children were vaccinated according to the Bangladesh Expanded Programme on Immunizations (EPI) vaccine schedule that was current at the time of the study, apart from polio and oral rotavirus vaccines, which were included in study interventions (Table 1).^9^

**Table 1:** Immunization schedule for the PROVIDE study

	Age (weeks)						
Vaccine	6	10	14	17	39	40	52
Pentavalent vaccine (diphtheria, whole-cell pertussis, tetanus, hepatitis B, *Haemophilus influenzae* type B)	X	X	X	–	–	–	–
Oral rotavirus vaccine (Rotarix)*	–	±	–	±	–	–	–
Trivalent oral polio vaccine (tOPV)*	X	X	X	–	±	–	X
Inactivated polio vaccine*	–	–	–	–	±	–	X
Measles + rubella	–	–	–	–	–	X	–
BCG (if not given at birth)	X	–	–	–	–	–	–

BCG = Bacillus Calmette–Guérin.

* Rotavirus and polio vaccines were given based on the study schedule.^4^^,^^6^ The vaccination schedule followed the Bangladesh Expanded Programme on Immunization.^9^

### Clinical and laboratory measures.

Basic sociodemographic assessment and anthropometric measurement were performed at enrollment and at prespecified scheduled follow-up according to the study protocol. Following enrollment, clinic visits occurred at 6, 10, 12, 14, 17, 18, 24, 39, 40, 52, 53, 65, 78, 91, and 104 weeks of life, with twice-weekly surveillance home visits and additional acute illness visits as indicated. Visits included review of any new concomitant medications, including antibiotics, which were prescribed or received outside of the study setting since the prior visit. Blood was collected at 53 weeks of age for measurement of plasma antibodies by Luminex targeting the following antigens: measles, pertussis toxin (PTX), pertussis filamentous hemagglutinin (FHA), pertussis pertactin (PRN), diphtheria toxoid (DTXD), tetanus toxoid (TTX), and Hib. Detailed methods for each assay, including the sources of antigens and critical reagents, methods of antigen conjugation, and issues related to assay validation and reproducibility have been previously published.^10^^–^^12^ Protective antibody levels for each target were determined according to previously published thresholds (Table 2).

**Table 2:** Protective antibody concentrations for select vaccines

Vaccine Antigen	Protective Antibody Level	Reference
Measles	≥0.12 IU/mL	13
PTX	≥8 EU/mL	14
FHA	≥8 IU/mL	3
PRN	≥8 IU/mL	15
DTXD	≥0.1 IU/mL	16
TTX	≥0.1 IU/mL	17
Hib	≥0.15 mcg/mL	17

EU = Equivalent Units; DTXD = diphtheria toxoid; FHA = filamentous hemagglutinin; Hib = *Haemophilus influenzae* type b; IU = International Units; PRN = pertussis pertactin; PTX = pertussis toxin; TTX = tetanus toxoid.

Data regarding antibiotic use were extracted from each participant’s chart after study completion via manual review of paper source documents, and clinic staff entered the data. Because the study clinic also served as the primary care clinic for participating families, virtually all prescriptions were obtained via the study clinic. All systemic antibiotic courses (oral, intravenous, or intramuscular), either prescribed by the study clinic or reported by the family as having been obtained from another source, were included in this analysis; no information on medication adherence was available other than family reports. Antibiotics were defined as narrow- or broad-based on spectrum of activity; amoxicillin and flucloxacillin were defined as narrow spectrum, with all other systemic antibiotics defined as broad-spectrum, based on expected activity against enteric Gram-negative and/or anaerobic bacteria (Table 3). The study population comprised all children who completed the parent study per protocol and had antibody concentration results at week 53 available for analysis.

**Table 3:** Antibiotics prescribed to study participants by week 53

Narrow Spectrum Antibiotics	Courses Prescribed, *n* (%) (*N* = 1,678)	Broad-Spectrum Antibiotics	Courses Prescribed, *n* (%) (*N* = 6,649)
Amoxicillin	1,159 (13.5)	Azithromycin	1,257 (14.7)
Flucloxacillin	519 (6.1)	Cefixime	2,059 (24.0)
		Cefpodoxime	9 (0.1)
		Ceftriaxone	186 (2.2)
		Cephradine	39 (3.3)
		Ciprofloxacin	1,089 (12.7)
		Cotrimoxazole	3 (0.03)
		Erythromycin	1,108 (12.9)
		Gentamicin	1 (0.01)
		Metronidazole	897 (10.5)
		Tetracycline	1 (0.01)

## STATISTICAL ANALYSES

Unadjusted and adjusted log-linear regression analyses were performed to evaluate the effect of antibiotic exposure, as measured by total antibiotic courses (as continuous variables), or days of antibiotics prescribed, on the natural log of antibody concentrations. Clinically pertinent covariates (height-for-age *z*-score at enrollment, weight-for-age *z*-score at enrollment, exclusive breastfeeding duration, delivery mode, birthplace, and sex) as well as the parent trial randomization assignments were included in the adjusted model. Antibiotic exposure through 14, 40, and 53 weeks of life was considered, although all vaccine antigens evaluated were administered by week 14, except for measles, which was administered at week 40. Lines of best fit and regression coefficients were calculated for each model. Coefficients from the model represent the difference in natural log antibody concentration per additional antibiotic course received and were then calculated to determine the estimated effect as percentage change in antibody concentration per antibiotic course. In addition, simple bivariate analysis was performed in which children were categorized dichotomously into high-exposure and low-exposure groups, defined as ≥21 days or <21 days of antibiotics, respectively, with natural log antibody concentrations compared between groups using independent sample *t*-tests. Children with or without antibody concentrations above the defined protective threshold were compared using Fisher’s exact test. Because multiple comparisons were undertaken, the Benjamini-Hochberg procedure was applied to all results to control the false discovery rate at 5%.^18^ All analyses were performed using R (v. 4.3.1; R Development Core Team).

## RESULTS

A total of 582 participants with evaluable data met inclusion criteria and were included in analysis. Most attrition was due to withdrawals or families moving out of the study area early during the study period, prior to completion of the EPI vaccine series, or visits that were missed or performed outside the specific window period, most often due to family travel.^6^ Basic sociodemographic information of the cohort is summarized in Table 4.

**Table 4:** Study population demographics

	*n* (%) or Mean ± SD
Total study subjects, *N*	582
Sex assigned at birth	
Male	304 (52)
Female	278 (48)
Mode of delivery	
Caesarian	135 (23)
Vaginal	447 (77)
Place of birth	
Home	148 (25)
Hospital	434 (75)
Breastfeeding status	
Contiguous weeks exclusively breastfed	16.3 ± 9.1
Participants with any breastfeeding at week 53	552 (95)
Anthropometry at enrollment (2–7 days old)	
Weight-for-age *z*-score	−1.3 ± 0.8
Length-for-age *z*-score	−0.9 ± 0.9
Weight-for-height *z*-score	−1.3 ± 0.9
Anthropometry at week 53	
Weight-for-age *z*-score	−1.1 ± 1.1
Length-for-age *z*-score	−1.4 ± 1.0
Weight-for-height *z*-score	−0.6 ± 1.0
Median (IQR) household income (1,000 taka)*	10 [7–15]
Median (IQR) maternal education (class years)	
No formal education	164 (28)
Class 1–5 (some primary)	217 (37)
Class 6–10 (some secondary)	173 (30)
Post-secondary	28 (5)

IQR = interquartile range; SD = standard deviation.

* In 2014, 1,000 taka was worth approximately USD $13.

As expected, this cohort was heavily prescribed a wide range of antibiotics. All antibiotics prescribed or reported by participants are listed in Table 3. Most major classes of antibiotics were represented, including penicillins, cephalosporins, macrolides, fluoroquinolones, sulfa drugs, tetracyclines, and anaerobic agents. Most (79.8%) of the prescribed antibiotics provided broad Gram-negative or anaerobic bacterial coverage, whereas 20.2% were narrow spectrum.

The median number of days on any antibiotic within the first year of life was 80 days (interquartile range [IQR]: 55 days), with 80% of antibiotic courses written for broad-spectrum antibiotics. By 14 weeks of life, only 7.6% of the cohort had not been prescribed antibiotics. By 40 weeks, 1% had no antibiotic use, and by 53 weeks of age, only 0.5% of study participants had no history of antibiotic exposure (*n* = 3). Study participants were prescribed a median of 62 days (IQR: 49 days) of broad-spectrum antibiotics in the first year of life, in comparison with 15 days (IQR: 17 days) of narrow-spectrum antibiotics. Most prescriptions (56%) were for respiratory illnesses, with approximately one-third (35%) for diarrheal illnesses.

Geometric mean antibody concentrations and the percentage of children with antibody concentrations above protective thresholds are listed in Table 5. Most children had protective antibody levels against measles, tetanus, and Hib (96%, 88%, and 91%, respectively), suggesting excellent response to these vaccine antigens. In contrast, most children had subprotective antibody levels against the three pertussis antigens tested and against diphtheria toxin, suggesting a less robust response to these antigens.

**Table 5:** Serum vaccine-induced antibody levels at 53 weeks of life and the percentage of study participants above protective antibody levels (*N* = 582)

Vaccine-Induced Plasma Antibody	Geometric Mean Concentration	95% CI	*n* (%) Above Protective Levels
Measles	0.74 IU/mL	(0.67 to 0.82)	561 (96)
PTX	5.93 EU/mL	(5.25 to 6.70)	244 (42)
FHA	5.49 EU/mL	(4.93 to 6.11)	189 (32)
PRN	3.87 EU/mL	(3.50 to 4.29)	145 (25)
DTXD	0.05 IU/mL	(0.05 to 0.06)	163 (28)
TTX	0.38 IU/mL	(0.35 to 0.42)	512 (88)
Hib	1.54 mcg/mL	(1.35 to 1.76)	529 (91)

EU = Equivalent Units; DTXD = diphtheria toxoid; FHA = filamentous hemagglutinin; Hib = *Haemophilus influenzae* type b; IU = International Units; PRN = pertussis pertactin; PTX =pertussis toxin; TTX = tetanus toxoid.

**Figure 1.**
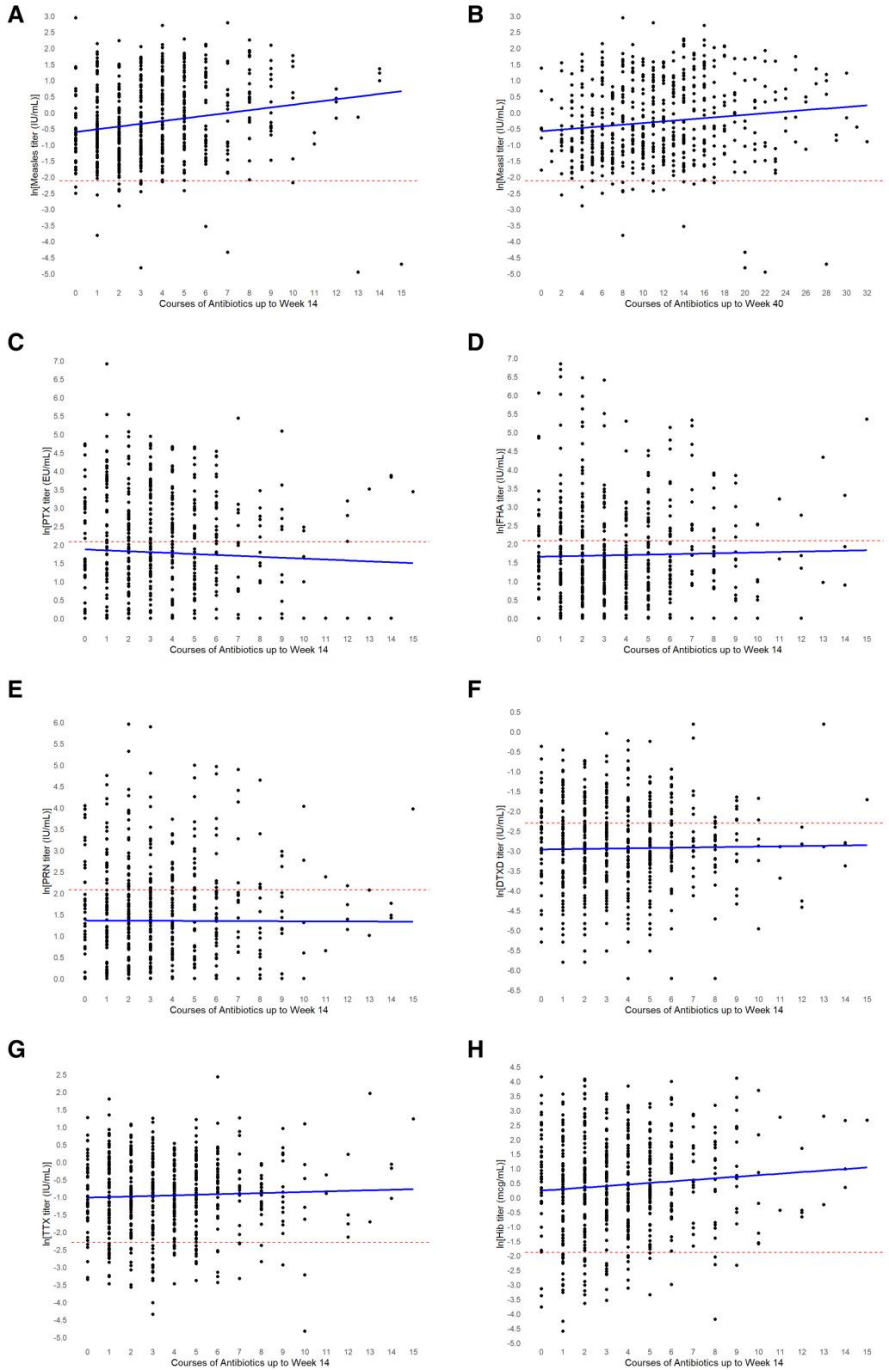
Natural log (Ln) of antibody concentrations by number of courses of antibiotics through the first 14 weeks of life and through 40 weeks for measles. (**A**) Ln Measles vs. antibiotics through 14 weeks; (**B**) Ln Measles vs. antibiotics through 40 weeks; (**C**) Ln PTX vs. antibiotics through 14 weeks; (**D**) Ln FHA vs. antibiotics through 14 weeks; (**E**) Ln PRN vs. antibiotics through 14 weeks; (**F**) Ln DTXD vs. antibiotics through 14 weeks; (**G**) Ln TTX vs. antibiotics through 14 weeks; (**H**) Ln Hib vs. antibiotics through 14 weeks. Blue line represents best fit by linear regression modeling. Red, dashed lines correspond to antibody-specific protective thresholds (Table 2).

The results of unadjusted and adjusted regression analyses for each vaccine antigen considering antibiotic exposure at 14 weeks of life (and 40 weeks for measles) are shown in Figure 1 and Table 6. No significant associations between antibiotic exposure and antibody concentrations were detected except for antibiotic courses at 14 and 40 weeks with measles concentration and at 14 weeks with Hib concentration. At 14 weeks, antibiotic courses were associated with an adjusted percent change of 9.09 for measles (95% CI: 5.13 to 12.75; *P* <0.001) and 5.65 for Hib antibody concentrations (95% CI: 1.01 to 11.63; *P* = 0.03); both results indicated modestly increased antibody concentrations with each additional antibiotic exposure. With antibiotic exposure up to 40 weeks, the percent change in concentration per antibiotic course for measles was 2.74 (95% CI: 1.01 to 4.08; *P* = 0.002). Weak negative associations were observed for PTX and DTXD concentrations with antibiotic exposures through weeks 40 and 53. However, because these represent additional antibiotic exposures after completion of the primary vaccine series for each, results were of unclear clinical significance. In bivariate analysis, no significant differences were observed in antibody concentrations in the high-exposure versus low-exposure groups for any antigen at any time point (see Supplemental Figures), and no significant differences were observed in the proportion of children with a protective antibody concentration against any antigen in the high-exposure versus low-exposure groups at any time point (data not shown).

**Table 6:** Results of log-linear regression modeling for courses of antibiotics at 14, 40, and 53 weeks vs. antibody concentrations. The adjusted model considers variables with potential effects on antibody concentrations*

Vaccine	% Change in Antibody Concentration per Course of Antibiotic (Unadjusted)	95% CI	*P*-Value	% Change in Antibody Concentration per Course of Antibiotic (Adjusted)	95% CI	*P*-Value
Measles						
Week 14	8.33	(5.13 to 12.75)	0.000009	9.09	(5.13 to 12.75)	0.000005^†^
Week 40	3.05	(1.01 to 4.08)	0.003	2.74	(1.01 to 4.08)	0.002^‡^
Week 53	0.30	(−1.00 to 2.02)	0.63	0.50	(−0.90 to 2.02)	0.47
PTX						
Week 14	−1.98	(−6.76 to 2.02)	0.28	−2.18	(−6.76 to 2.02)	0.35
Week 40	−1.98	(−3.92 to 0.30)	0.02	−2.08	(−3.92 to −0.10)	0.04^‡^
Week 53	−1.98	(−2.96 to −0.10)	0.03	−1.69	(−2.96 to −0.04)	0.04^‡^
FHA						
Week 14	1.01	(−2.96 to 5.13)	0.52	1.41	(−2.96 to 5.13)	0.5
Week 40	−1.00	(−1.00 to −1.98)	0.48	−0.50	(−1.98 to 1.01)	0.62
Week 53	−0.30	(−1.98 to 1.01)	0.62	−0.20	(−1.98 to 1.01)	0.82
PRN						
Week 14	−0.20	(−3.92 to 4.08)	0.92	−0.40	(−3.92 to 3.05)	0.82
Week 40	−1.00	(−1.98 to 1.01)	0.52	−0.60	(−1.98 to 1.01)	0.51
Week 53	−0.40	(−1.98 to 1.01)	0.53	−0.40	(−1.98 to 1.01)	0.54
DTXD						
Week 14	1.01	(2.96 to 4.08)	0.64	0.80	(−2.96 to 4.08)	0.64
Week 40	−1.98	(−2.96 to 0.00)	0.05	−1.49	(−2.96 to 0.10)	0.06
Week 53	−1.00	(−2.96 to 0.10)	0.02	−1.49	(−2.96 to −0.20)	0.02^‡^
TTX						
Week 14	2.02	(−1.98 to 5.13)	0.34	1.21	(−1.98 to 5.13)	0.42
Week 40	−0.30	(−1.98 to 1.01)	0.67	−0.50	(−1.98 to 1.01)	0.5
Week 53	−1.00	(−1.98 to 1.01)	0.37	−0.70	(−1.98 to 0.40)	0.24
Hib						
Week 14	5.13	(0.30 to 10.52)	0.04	5.65	(1.01 to 11.63)	0.03^‡^
Week 40	2.02	(−0.40 to 4.08)	0.11	1.92	(−0.30 to 4.08)	0.1
Week 53	1.01	(−1.00 to 2.02)	0.48	0.70	(−1.00 to 3.05)	0.44

DTXD = diphtheria toxoid; FHA = filamentous hemagglutinin; Hib = *Haemophilus influenzae* type b; TTX = tetanus toxoid.

* Variables in adjusted model: height-for-age *z*-score, weight-for-age *z*-score, sex, number of days exclusively breastfed, delivery status (cesarean vs. vaginal), birthplace (home vs. hospital), rotavirus vaccine randomization arm, polio vaccine randomization arm.

^†^ Statistically significant (*q*-value = 0.0003) after correction for false discovery rate.

^‡^ Not statistically significant after correction for false discovery rate.

After 5% false discovery rate correction, only measles antibody concentrations according to week 14 antibiotic exposure remained statistically significant (*q*-value = 0.0003).

## DISCUSSION

Vaccinations are an integral part of comprehensive pediatric care to reduce child mortality and promote healthy growth and development. In Bangladesh and other LMIC settings with extraordinary rates of diarrheal^18^ and respiratory infections^19^ in young children, antibiotics are both lifesaving but also very easily accessible, making appropriate antibiotic stewardship a significant challenge. It is thus imperative to better understand the long-term effects of antibiotic treatment that may cause harm in pediatric populations. The results of this work show that in infants from Dhaka, Bangladesh, requiring antibiotic treatment, the degree of antibiotic exposure in infancy did not appear to clinically impact parenteral vaccine-induced antibody concentrations against FHA, PRN, Hib, DTXD, and tetanus toxin. After false discovery rate correction, statistically significant associations were only observed between number of courses of antibiotics at 14 weeks and measles antibody concentration. Contrary to our expectations, each additional course of antibiotics was associated with a slight increase in antibody concentration. Although this finding was highly statistically significant, the clinical significance is likely very limited, given that almost all children had protective antibody levels against measles and that this association diminished over time with additional antibiotic exposures by weeks 40 and 53. In addition, the wide range of antibody concentrations observed across all levels of antibiotic exposure, not only for measles but for all antigens tested, suggest that antibiotics were a relatively poor predictor of final antibody concentration. For all tested antigens, no significant differences were observed in antibody concentration or the frequency of achievement of a protective antibody level when children were categorized as having had high versus low antibiotic exposure.

These results contrast with a recent publication by Chapman et al. among U.S. children, which demonstrated decreased serum antibody concentrations in antibiotic-exposed versus unexposed children, with a consistent dose–response relationship between antibiotic courses and associated decreases in antibody concentrations observed, even to sub-protective levels.^3^ In that study, children with any antibiotic exposure were shown to have diminished concentrations to DTaP, Hib, IPV, and pneumococcal (PCV) vaccinations. Following booster doses of these vaccines, antibody levels to DTaP were reduced by 18.1%, Hib by 21.3%, IPV by 18.9%, and PCV by 12.2% for each antibiotic course the child received.^3^ Based on these results, the authors strongly suggested caution about overprescribing antibiotics to young children due to the potential reduction in vaccine responses. In contrast, our data did not indicate a clinically significant association between antibiotic courses and antibody concentrations. We chose to compare number of courses of antibiotics rather than days of antibiotics because we found the two were highly correlated and believed the course number to be a more accurate measure due to the lack of available adherence data. In addition, there are significant differences between our cohort and that studied by Chapman et al., including profound differences in the degree of antibiotic exposure; in background demographic, geographic, racial, and socioeconomic variables; and in the vaccine schedules and products used.

We found that our study population had much higher numbers of antibiotic prescriptions in the first year of life in comparison with pediatric populations in other settings. An astonishing 99% of our study population had at least one antibiotic prescription during infancy, in comparison with 20–40% in prior studies of antibiotic prescription among children from the United States and Denmark.^20^^,^^21^ In a different U.S. setting, even by year 2 of life, only up to 70% of young children had received a single antibiotic course.^22^ Most other LMICs also demonstrate rates of antibiotic use closer to reported U.S. averages, meaning that our study population experienced an unusually high rate of antibiotic prescriptions.^23^ In addition, due to the high rates of diarrheal illnesses in this setting, it is likely that a relatively higher proportion of broad-spectrum antibiotics, specifically to target enteric pathogens, were prescribed to this patient population in comparison with children in high-income settings, where a larger proportion of prescriptions would be for respiratory tract infections. For example, one recent meta-analysis of outpatient antibiotic prescriptions for children in high-income settings found that prescriptions for treatment of gastroenteritis had the highest proportion of expanded-spectrum agents prescribed, but in general (based on few studies) the lowest rate of antibiotic prescriptions written, in comparison with other indications.^24^

This study had several limitations. First, this was a secondary analysis that the parent trial was not primarily designed to conduct and is thus exploratory in nature. Next, vaccine-induced antibody concentrations were only measured at 53 weeks of age, many weeks after the vaccines were administered. Significant waning of antibody levels may occur over time, which may explain the high proportion of study participants who were below protective levels for pertussis and diphtheria antigens. Because all participants received a dose of OPV at week 52 as an oral challenge dose for the polio arm of the parent trial, it is possible that active immune response to OPV could have modulated the observed antibody results in the week 53 sample. We theorize that any effect is likely to be minimal, however, because 1) all participants had previously completed a primary polio vaccine series, 2) OPV infection is asymptomatic, and 3) only 36% of children shed culturable virus following this challenge dose, meaning most children did not have a successful vaccine take of this dose.^8^ Next, due to the wide window periods for study visits later during infancy, there was variability in when the week 53 visit was conducted for study participants and therefore when serum was drawn for antibody concentration analysis. Additionally, the reliance on family reports for antibiotic use outside the clinic could introduce recall bias in the data.

However, a strength of the study is that prescription information was fairly complete for all participants, given that the study site served as the primary care clinic for study participants. Due to the high rates of antibiotic use in this population, very few participants (only 3 out of 582) had no antibiotic exposure in the first year of life, meaning that we were unable to directly compare a group that had not been exposed to any antibiotics to those who had. Even by the week 14 visit, only 7.5% of the cohort had not been prescribed a course of antibiotics. It is possible that the greatest effect of antibiotic use occurs with the first exposure, as suggested by the previous report from Chapmen et al. in U.S. children, and could potentially explain the lower responses observed against pertussis and diphtheria antigens; unfortunately, however, we were unable to directly assess for this effect.^3^

Finally, immune correlates of protection for many of the antigens tested remain controversial. For pertussis, pertussis toxin antibodies have been the most well-characterized, with a cutoff of 5 EU/mL proposed as a minimum protective value.^14^ We chose a slightly higher cutoff of 8 EU/mL to remain consistent in comparisons with the other recent report by Chapman et al., but if applying the minimum cutoff, our estimates would improve slightly to 51% of children having a protective concentration. It remains unclear at what levels antibodies against the pertussis antigens FHA and PRN further contribute to clinical protection. For anti-diphtheria toxoid and anti-tetanus toxin antibodies, two cutoffs to define protection can be applied: 0.01 IU/mL and 0.1 IU/mL, corresponding to minimum protection against any disease versus robust protection. The lower threshold has typically been validated in the setting of functional neutralization assays, with the higher threshold preferred when measured using binding assays.^17^ Therefore, we chose the latter, more conservative protective cutoff for our analysis to be as inclusive as possible for study participants with sub-protective levels. However, if applying the lower cutoff to assess for any evidence of protection, the percentage of study participants with potentially protective levels against diphtheria toxin and tetanus toxin at 53 weeks of age would have been 93% for diphtheria and 99% for tetanus. Overall, we believe a reasonable interpretation of our results is that vaccine response to tetanus, Hib, and measles were excellent, marginal to pertussis, and low for diphtheria. While response to diphtheria toxoid was weaker than expected, almost all children in this cohort did have evidence of some degree of response to the vaccine.

Direct comparisons of the proportion of children who achieved seroprotective levels and the antibody concentrations achieved from our data to studies from high-income countries (HICs) in general are also limited by to the delayed time of antibody measurement performed here, in comparison with typical immunogenicity studies. Despite this, detectable rates of measles antibody seroconversion after a single dose at 9 months was actually higher in this population than typically expected for an LMIC setting^25^ and compared favorably with the 95% expected rate of antibody response following immunization at 12 months in children from HICs.^26^ Similarly, antibody responses to Hib and tetanus were similar to those seen following primary vaccination in HICs, where nearly all infants are expected to develop protective antibody levels to tetanus^27^ and 95% to Hib^28^ after a three-dose primary vaccine series. In contrast, our observed antibody concentrations were far lower for diphtheria, which reliably induces protective antibody levels in nearly all vaccinated infants in HICs following the three-dose primary series, and for all pertussis antigens, against which most infants develop antibody seroresponse after three doses,^27^ although whether this was due to primary vaccine failure versus rapid antibody waning in our cohort could not be determined given the limitation in specimen timing.

Although the precise mechanisms through which antibiotics would potentially impact parenteral vaccine responsiveness remain elusive, an attractive hypothesis is that antibiotic exposure may disrupt the complex interplay between the developing microbiome and adaptive immune responses.^23^^,^^29^ Most studies have inferred an effect based on the relationship between gut microbiome and its relationship with vaccine immunogenicity, but few have evaluated the effects of antibiotic exposure directly.^30^^,^^31^ Given the lack of meaningful differences in our study’s primary outcome measures, analysis of the gut microbiome in these infants was not further pursued, because it is unlikely that any microbiome measures could be further implicated in a mechanistic pathway in this cohort. However, for this reason, whether the first antibiotic exposure could have had the greatest influence on both microbiome development and vaccine response in this population remains unconfirmed.

## CONCLUSION

In summary, exposure to antibiotics in the first year of life did not seem to significantly impact antibody concentrations in response to several common parenteral childhood vaccinations in children from Dhaka, Bangladesh. It was reassuring that higher levels of antibiotic exposure in this study population did not appear to be directly associated with lower levels of parenteral vaccine-induced antibodies, which is important information for the treatment of children in low-resource settings. However, these findings highlight the need for further research to unravel the nuanced dynamics at play, particularly in children with any antibiotic exposure versus none, and the high degree of antibiotic exposure observed here suggests that greater efforts to promote more prudent antibiotic use in high-risk populations to limit global antimicrobial resistance and other adverse effects remains a critical need.

## Supplemental Materials

10.4269/ajtmh.25-0511 Supplemental Materials
