# Supplementary material for: Parenteral Vaccine-Induced Antibody Levels in Children with Heavy Antibiotic Exposure in Dhaka, Bangladesh
Source: Am J Trop Med Hyg. 2026 Jun 4;115(2):340–7. doi: 10.4269/ajtmh.25-0511 (PMC13447802; doi:10.4269/ajtmh.25-0511)

Supplementary Figure 1. Natural log of measles antibody concentrations by high (>21 days) or low exposure (≤21 days) to antibiotics through the first 14, 40, and 53 weeks of life. Red points and error bars represent the geometric mean concentration with 95% CI, respectively. None of the tested differences were statistically significant. Dashed lines represent protective thresholds for each antigen.

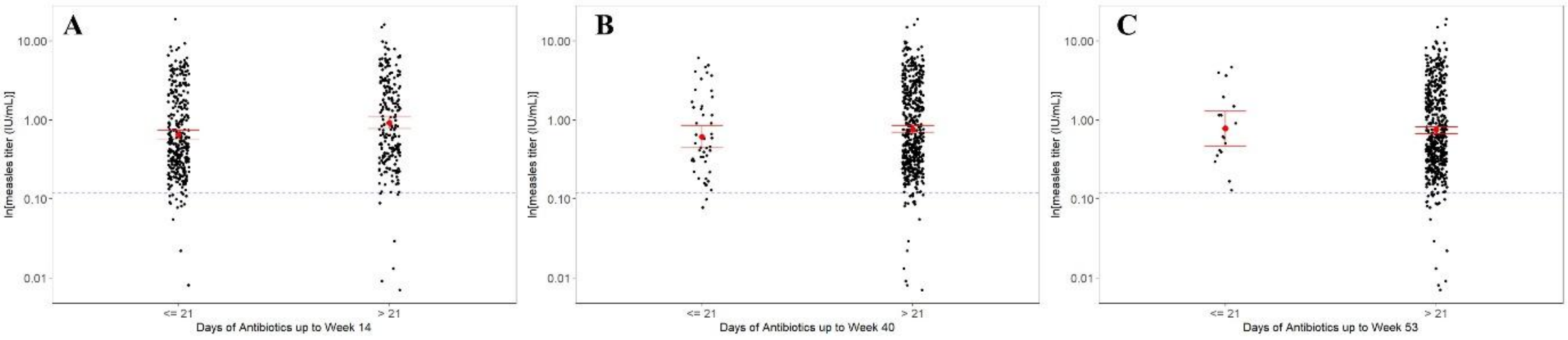

Supplementary Figure 2. Natural log of PTX antibody concentrations by high (>21 days) or low exposure (≤21 days) to antibiotics through the first 14, 40, and 53 weeks of life. Red points and error bars represent the geometric mean concentration with 95% CI, respectively. None of the tested differences were statistically significant. Dashed lines represent protective thresholds for each antigen.

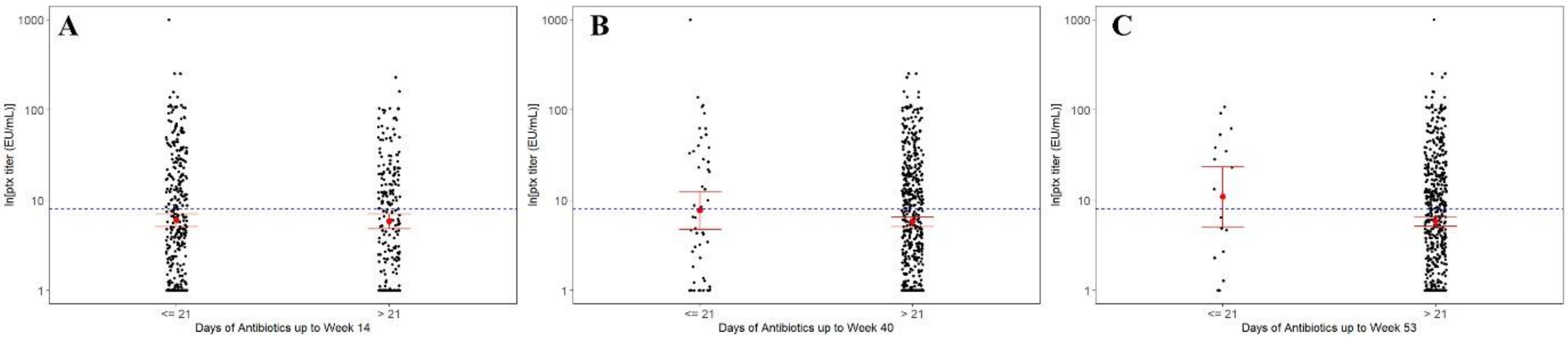

Supplementary Figure 3. Natural log of FHA antibody concentrations by high (>21 days) or low exposure (≤21 days) to antibiotics through the first 14, 40, and 53 weeks of life. Red points and error bars represent the geometric mean concentration with 95% CI, respectively. None of the tested differences were statistically significant. Dashed lines represent protective thresholds for each antigen.

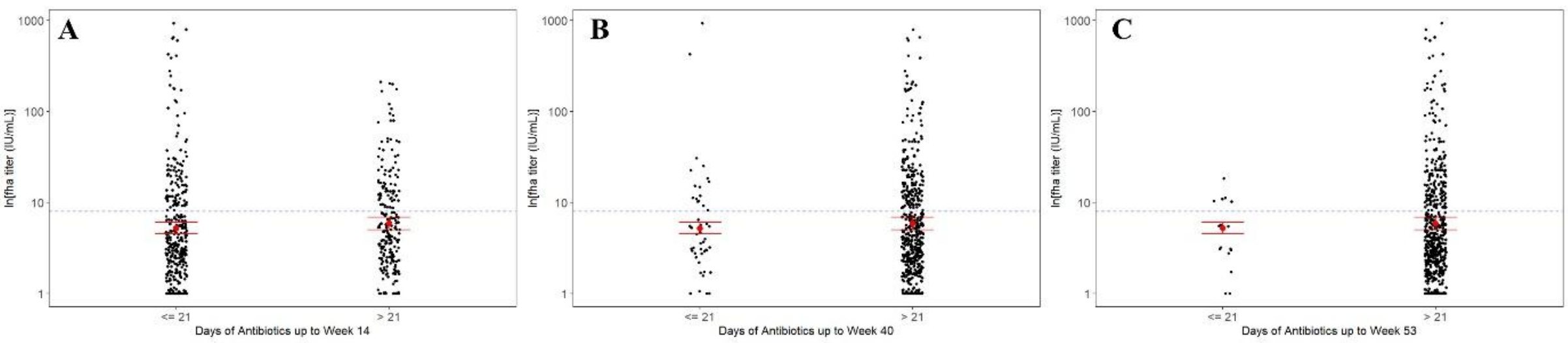

Supplementary Figure 4. Natural log of PRN antibody concentrations by high (>21 days) or low exposure (≤21 days) to antibiotics through the first 14, 40, and 53 weeks of life. Red points and error bars represent the geometric mean concentration with 95% CI, respectively. None of the tested differences were statistically significant. Dashed lines represent protective thresholds for each antigen.

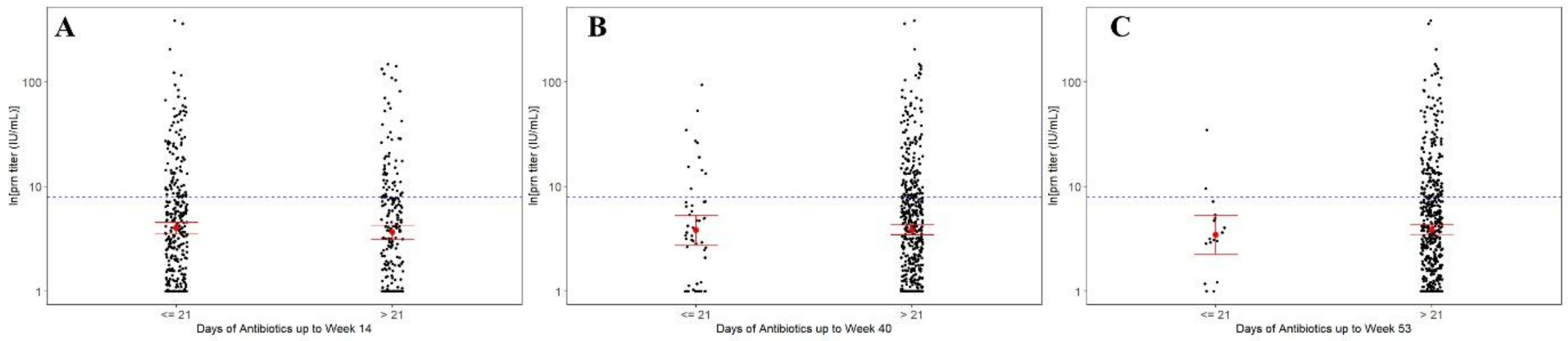

Supplementary Figure 5. Natural log of DTXD antibody concentrations by high (>21 days) or low exposure (≤21 days) to antibiotics through the first 14, 40, and 53 weeks of life. Red points and error bars represent the geometric mean concentration with 95% CI, respectively. None of the tested differences were statistically significant. Dashed lines represent protective thresholds for each antigen.

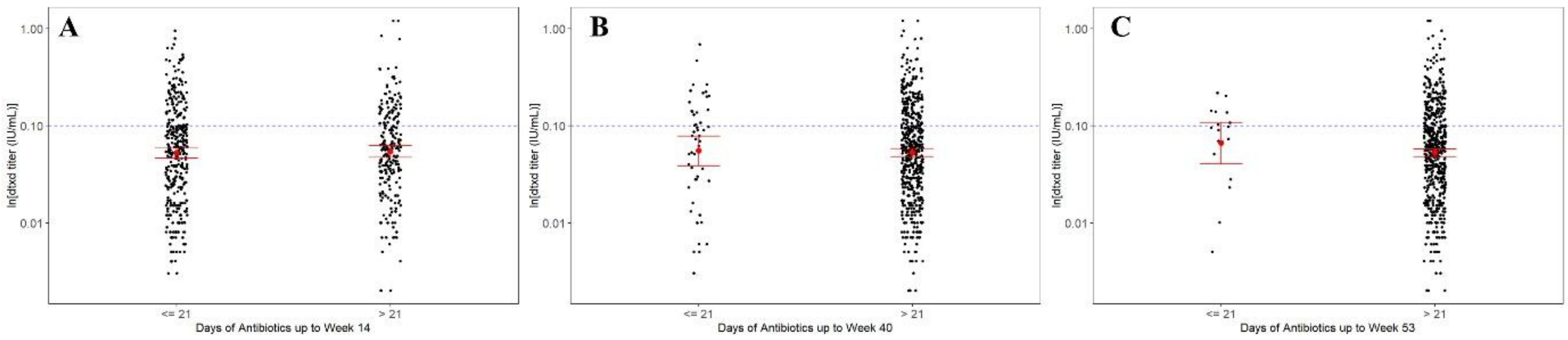

Supplementary Figure 6. Natural log of TTX antibody concentrations by high (>21 days) or low exposure (≤21 days) to antibiotics through the first 14, 40, and 53 weeks of life. Red points and error bars represent the geometric mean concentration with 95% CI, respectively. None of the tested differences were statistically significant. Dashed lines represent protective thresholds for each antigen.

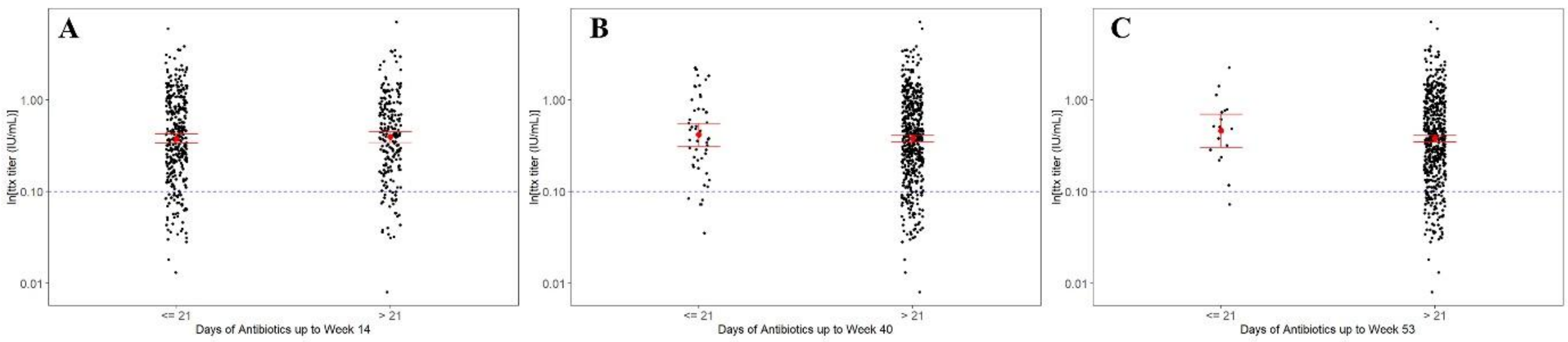

Supplementary Figure 7. Natural log of Hib antibody concentrations by high (>21 days) or low exposure (≤21 days) to antibiotics through the first 14, 40, and 53 weeks of life. Red points and error bars represent the geometric mean concentration with 95% CI, respectively. None of the tested differences were statistically significant. Dashed lines represent protective thresholds for each antigen.

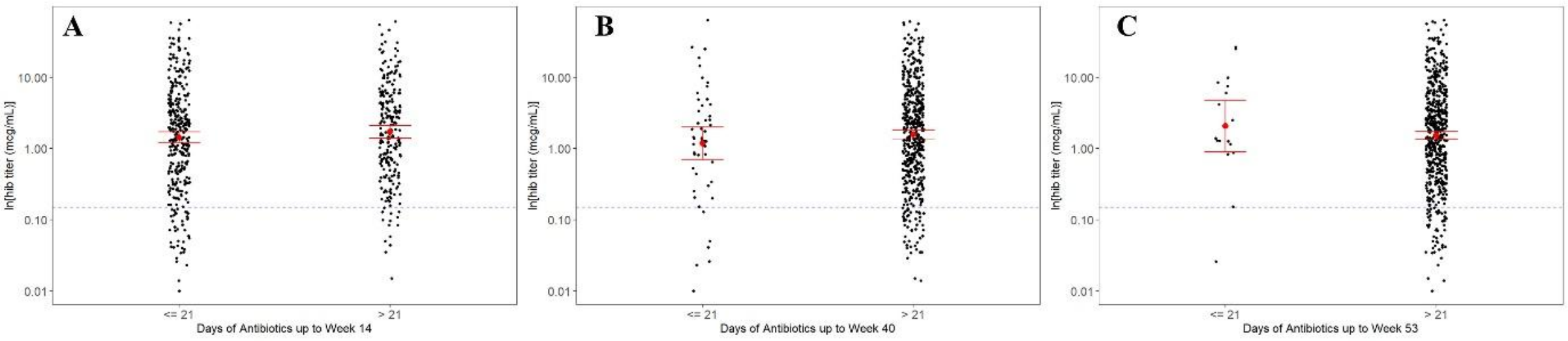

Supplement: Supplementary file 1 [file tpmd250511.SD1.pdf]
